# Mechanisms of BPA Degradation and Toxicity Resistance in *Rhodococcus equi*

**DOI:** 10.3390/microorganisms11010067

**Published:** 2022-12-26

**Authors:** Kejian Tian, Yue Yu, Qing Qiu, Xuejian Sun, Fanxing Meng, Yuanping Bi, Jinming Gu, Yibing Wang, Fenglin Zhang, Hongliang Huo

**Affiliations:** 1 School of Environment, Northeast Normal University, No. 2555 Jingyue Avenue, Changchun 130117, China; 2 Jilin Province Water Resources and Hydropower Consultative Company of P.R. China, Changchun 130021, China; 3 School of Life Sciences, Northeast Normal University, No. 5268, Renmin Main Street, Changchun 130024, China; 4 Jilin Province Laboratory of Water Pollution Treatment and Resource Engineering, Changchun 130117, China; 5 Northeast China Low Carbon Water Pollution Treatment and Green Development Engineering Research Center, Changchun 130117, China

**Keywords:** Bisphenol A, *Rhodococcus*, biodegradation, toxicity resistance

## Abstract

Bisphenol A (BPA) pollution poses an increasingly serious problem. BPA has been detected in a variety of environmental media and human tissues. Microbial degradation is an effective method of environmental BPA remediation. However, BPA is also biotoxic to microorganisms. In this study, *Rhodococcus equi* DSSKP-R-001 (R-001) was used to degrade BPA, and the effects of BPA on the growth metabolism, gene expression patterns, and toxicity-resistance mechanisms of *Rhodococcus equi* were analyzed. The results showed that R-001 degraded 51.2% of 5 mg/L BPA and that 40 mg/L BPA was the maximum BPA concentration tolerated by strain R-001. Cytochrome P450 monooxygenase and multicopper oxidases played key roles in BPA degradation. However, BPA was toxic to strain R-001, exhibiting nonlinear inhibitory effects on the growth and metabolism of this bacterium. R-001 bacterial biomass, total protein content, and ATP content exhibited V-shaped trends as BPA concentration increased. The toxic effects of BPA included the downregulation of R-001 genes related to glycolysis/gluconeogenesis, pentose phosphate metabolism, and glyoxylate and dicarboxylate metabolism. Genes involved in aspects of the BPA-resistance response, such as base excision repair, osmoprotectant transport, iron-complex transport, and some energy metabolisms, were upregulated to mitigate the loss of energy associated with BPA exposure. This study helped to clarify the bacterial mechanisms involved in BPA biodegradation and toxicity resistance, and our results provide a theoretical basis for the application of strain R-001 in BPA pollution treatments.

## 1. Introduction

Although BPA is one of the most widely used chemicals in the world, it has significant toxic effects on human health. Studies have shown that BPA blocks the estrogen response by competing with E2 [1]. BPA also binds to androgen receptors, leading to androgen-dependent gene regulation disorders [2] and causing reproductive, developmental, and metabolic diseases [1,3,4,5]. BPA is carcinogenic and mutagenic, inducing prostate cancer [6], breast tumors [7], and ovarian cancer [8]. BPA can destroy immune-related signaling pathways [9], damage the immune system [10], and even induce increases in the contents of T helper cell type 1 (Th1) and Th17 in humans, leading to various cancers and autoimmune diseases (e.g., type 1 diabetes) [11].

BPA also causes serious harm to lower organisms such as algae, fish, and amphibians [12]. Even at low concentrations, BPA can inhibit algal growth, reproduction, and photosynthesis [13]. The median lethal concentration (LC50) of BPA is only 6.8–17.9 mg/L for fish, and the LC50 for amphibians is even lower [14].

At present, the average daily intake of BPA in the global population is 38.78 ng/kg bw/day for adults and 51.74 ng/kg bw/day for children [15]. Although there is no uniform standard for the minimum harmful concentration of BPA for humans, long-term exposure to BPA undoubtedly has negative health effects.

Microbial degradation is an effective method to relieve BPA pollution [16]. Bacteria that degrade BPA have been isolated from soil, water, WWTPs, and other environmental media [16]. For example, *Cupriavidus basilensis* SBUG 290, isolated from compost soil, degraded 78% of 0.26 mM BPA after induction [17], while *Bacillus* sp. AM1, isolated from infant feces, removed 84.68% of 25 μg/L BPA [18]. Finally, *Bacillus megaterium* ISO-2, isolated from polycarbonate industrial wastewater, completely removed 5 mg/L BPA within 72 h [19]. However, the toxicity of BPA can significantly inhibit microbial metabolism and growth [20]: BPA not only inhibits the bioactivity and organic degradation capacity of activated sludge but can also alter microbial community structure, decreasing the abundance of functional bacteria involved in water purification [21,22]. Indeed, this is one of the reasons why WWTPs cannot completely remove BPA from wastewater. In addition, BPA can significantly reduce microbial activity and the growth of microorganisms in the soil, change microorganismal community structure, and inhibit the activity of certain enzymes [20,23]. Microorganisms adapt to BPA-associated biological stress by upregulating genes encoding xenobiotic degradation proteins, flagellins, and biofilm-related proteins [24]. *Rhodococcus equi* R-001 has a good degradation capacity and a high tolerance for BPA toxicity. However, the toxic effects of BPA on *Rhodococcus* and the molecular mechanisms of resistance and degradation underlying the response of *Rhodococcus* to BPA stress remain unclear.

Therefore, two important foci of BPA bioremediation studies are to screen more efficient BPA-degrading bacteria and to analyze the mechanisms underlying the resistance of bacterial strains to BPA toxicity. In this study, we investigated the toxic effects of BPA on microorganisms and the transcriptomic response to BPA stress using *Rhodococcus equi* DSSKP-R-001 as an exemplar strain. The resistance mechanisms underlying the response of R-001 to BPA toxicity were then further analyzed. This study expanded the known roster of microbial species that degrade BPA, helped to clarify the molecular mechanisms underlying microbial tolerance of BPA, and provided a theoretical basis for the application of R-001 in BPA pollution treatment.

## 2. Materials and Methods

### 2.1. Strains and Chemicals

*Rhodococcus equi* DSSKP-R-001 was screened and purified in our laboratory and stored in the China Microbial Species Preservation Center (CGMCC No. 12392). BPA (Product No. B108653-50g) was purchased from Shanghai Aladdin Biochemical Technology Co., Ltd., Shanghai, China (https://www.aladdin-e.com/zh_cn/ (16 March 2022)); and ethyl acetate (34858), methanol (1.06035), and acetonitrile (1.00029) were purchased from Sigma-Aldrich (https://www.sigmaaldrich.cn/CN/zh (16 March 2022)). Bacterial RNA extraction kits were purchased from Tiangen Biotech Co., Ltd. (Beijing, China).

### 2.2. R-001 Degrades BPA

Bacterial suspensions (initial OD_600_ of 1.0) were added to a mineral basal medium supplemented with different concentrations of BPA at an initial ratio of 4% (by volume). The inoculated media were placed in a constant-temperature shaking incubator at 30 °C with shaking at 120 rpm and incubated for 2, 4, 24, 48, 96, or 120 h. The initial concentrations of BPA were 5 mg/L, 10 mg/L, 15 mg/L, 20 mg/L, 30 mg/L, 40 mg/L, and 50 mg/L, and three parallel samples were analyzed per group.

### 2.3. Detection Methods

High performance liquid chromatography (HPLC), performed using a Zorbax Eclipse Plus C18 column (150 × 4.6 mm, 3.5 mm), was used to detect the concentration of the remaining substrate in the reaction system. The ratio of acetonitrile to water in the mobile phase (*v*/*v*) was 1:1, and the flow rate was 0.8 mL/min. The detector wavelength was 281 nm, the column temperature was 30 °C, and the injection volume was 10 μL. An Agilent liquid chromatography G6400 series triple quadrupole mass spectrometer was used to analyze the degradation intermediate product of BPA. The mobile phase was methanol:water (*v*/*v*) = 1:1, the detection wavelength was 281 nm, the flow rate was 0.8 mL/min, the injection volume was 2 μL, and the oven temperature was maintained at 30 °C. The electrospray ionization (ESI) method was used in positive and negative ion mode scans in the range of 50–600 Da. The ESI source conditions were as follows: source temperature of 80 °C, desolvation temperature of 250 °C, capillary voltage of +3 kV, and a cone gas flow rate of 50 L/h. The protein concentration was detected with the BCA method using the BCA Protein Quantification Kit (E112-01). The ATP content of the bacteria was detected with the phosphomolybdic acid colorimetric method using an ATP content determination kit (G0815W96).

### 2.4. Transcriptome Sequencing

The concentrations of BPA in the experimental group were 5 mg/L (group A1), 15 mg/L (group A2), and 40 mg/L (group A3), and the glucose concentration in the control group was 5 mg/L (group B1). After 120 h of culture, the samples were centrifuged at 4 °C and 10,000 rpm/min for 5 min, and the supernatant was discarded. Thalli were collected in a 1.5 mL RNase-free centrifuge tube, sealed, immediately transferred to liquid nitrogen, and flash-frozen for 30 min. Sequencing was performed on an Illumina Hiseq Platform. Genes were considered significantly differentially expressed when qValue was ≤0.05 and |log_2_^FoldChange^| was ≥1. The raw sequence data has been submitted to the NCBI database (accession number PRJNA842860). The raw expression levels of the significantly differentially expressed genes (DEGs) from the transcriptome analysis are shown in Supplementary Tables S1–S5.

### 2.5. qPCR Analysis of the BPA-Degrading Genes

To further investigate the expression levels of the BPA-degrading genes, qPCR analysis was performed. The culture conditions were described in Section 2.4. Cultures exposed to BPA concentration groups q1 (5 mg/L), q2 (15 mg/L), and q3 (40 mg/L), respectively. RNA extraction and qPCRs were performed as previously described [25], and the primers used are shown in Supplementary Table S6. Gene expression levels in each sample were calculated relative to the expression levels of the *recA* reference gene using the 2^−ΔΔCt^ quantification method.

### 2.6. Bioinformatics Analysis

The BLAST platform was used to analyze the key genes associated with BPA degradation. The amino acid sequences of previously reported BPA-degrading enzymes were aligned, including cytochrome P450 monooxygenase (accession no. WP_106851580.1, derived from *Rhodococcus equine* DSSKP-R-001; accession no. OMQ03826.1, derived from *Bacillus* sp. GZB; and accession no. BAG15884.1, derived from *Sphingomonas bisphenolicum* AO1) and laccase (accession no. APF29085.1, derived from *Bacillus* sp. GZB).

## 3. Results and Discussion

### 3.1. BPA Degradation by R-001

**Figure 1:**
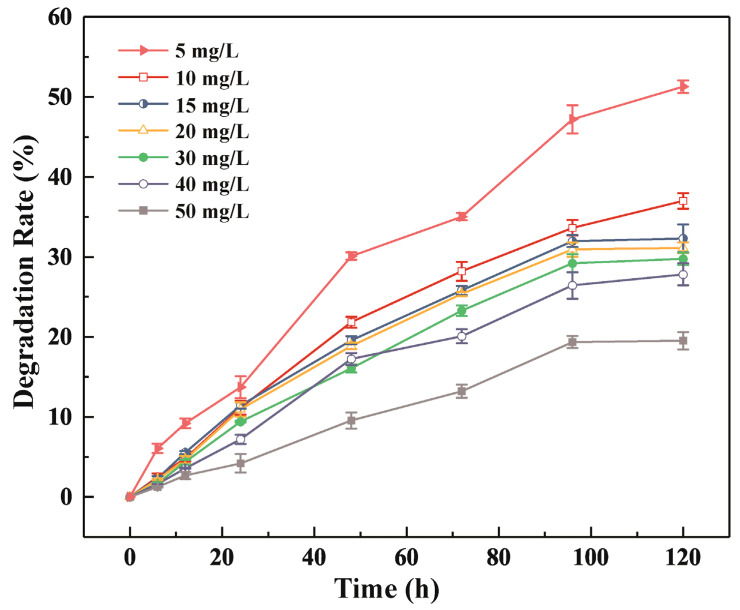
Degradation of BPA by strain R-001 at different concentrations.

*Rhodococcus equi* DSSKP-R-001 had a higher BPA degradation rate at low concentrations than at high concentrations, and the degradation rate decreased as the concentration of BPA increased. At a BPA concentration of 5 mg/L, the degradation rate was 51.2%, and when the BPA concentration was increased to 40 mg/L, the degradation rate was 28.2%. Above this concentration, the degradation rate decreased significantly: the degradation rate was only 19.52% at a BPA concentration of 50 mg/L (Figure 1). These results demonstrated that R-001 can use BPA as the sole carbon source and degrade it efficiently. However, 40 mg/L may be the maximum concentration of BPA that R-001 can tolerate while maintaining its degradation function. In comparison, *Bacillus pumilus* BP-2CK, BP-21DK, and BP-22DK must be supplemented with other nutrients during BPA degradation [26]. A previous study on five probiotics found that the highest rate of BPA degradation (at a BPA concentration of 50 µg/L) was only 51.9% [27], and the removal of 1.7 mg BPA (0.15 mM) by *Cupriavidus basilensis* JF1 took 144 days [28]. R-001 showed comparatively excellent BPA degradation performance with respect to both BPA concentration and BPA degradation time. These results suggested that R-001 has a good potential utility for BPA degradation and has theoretical value for further analysis.

### 3.2. Toxic Effects of BPA on the Growth and Metabolism of R-001

**Figure 2:**
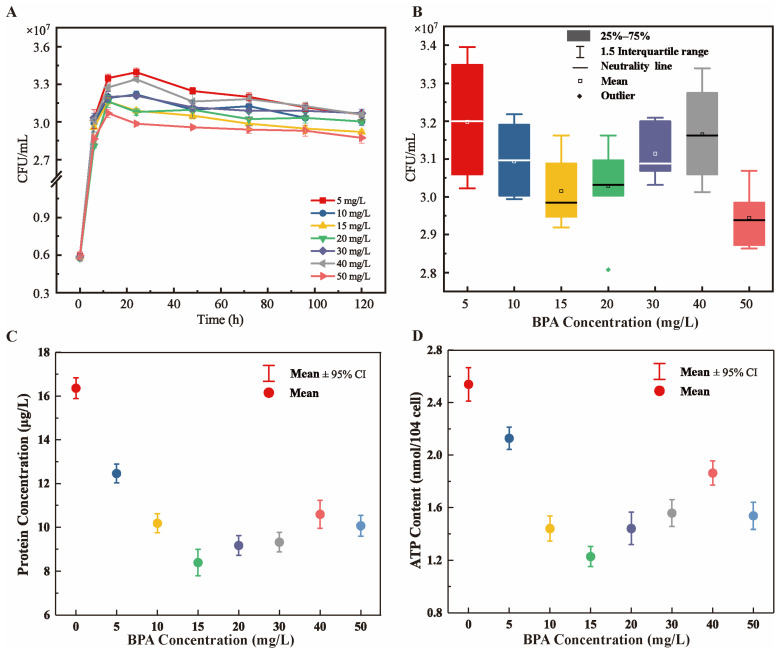
Inhibition of R-001 by different concentrations of BPA. (**A**) Strain growth. (**B**) Variations in CFU value. (**C**) Changes in protein concentration. (**D**) Changes in ATP content.

The cytotoxicity of BPA and the antitoxic function of R-001 were very obvious when R-001 was cultured with BPA. Different concentrations of BPA significantly affected the bacterial biomass of R-001. At BPA concentrations of 5–15 mg/L, the bacterial biomass of R-001 continued to decrease, and at BPA concentrations of 15–40 mg/L, the bacterial biomass of R-001 increased. At BPA concentrations above 40 mg/L, the bacterial biomass of R-001 decreased again. In general, the bacterial biomass exhibited a “V” shape as the BPA concentration increased from 5 mg/L to 40 mg/L (Figure 2A,B).

Total intracellular protein concentration and ATP content effectively reflect organismal growth and metabolism [29]. Under BPA stress, protein concentration and ATP content in R-001 showed a V-shaped trend. At a BPA concentration of 15 mg/L, protein concentration and ATP content were minimized (8.447 μg/mL and 1.21 nmol/104 cells, respectively). At a BPA concentration of 50 mg/L, ATP content decreased significantly, suggesting that BPA concentration may have exceeded the tolerance range of strain R-001 (Figure 2C,D). The results indicated that the inhibitory effects of BPA toxicity on R-001 were nonlinear. Similarly, *Roseobacter* sp. AzwK-3b grows normally at a BPA concentration of 9 μM, but BPA concentrations greater than 18 μM can inhibit the strain. Moreover, exposure to 18 and 44 μM BPA reduced the OD_600_ value of strain AzwK-3b by about 20% and 67%, respectively [30]. Other studies have shown that BPA reduces the protein content of microorganisms [31] and even the ATP levels in human cells [32]. Organisms can also activate antioxidant mechanisms to trigger retrograde signal transduction and enhance BPA tolerance [33]. Our results also showed that the protein and ATP contents of strain R-001 tended to increase as BPA concentrations increased from 15 mg/L to 40 mg/L.

### 3.3. Inhibitory Effects of BPA on the Expression Profiles of Metabolic Genes

**Figure 3:**
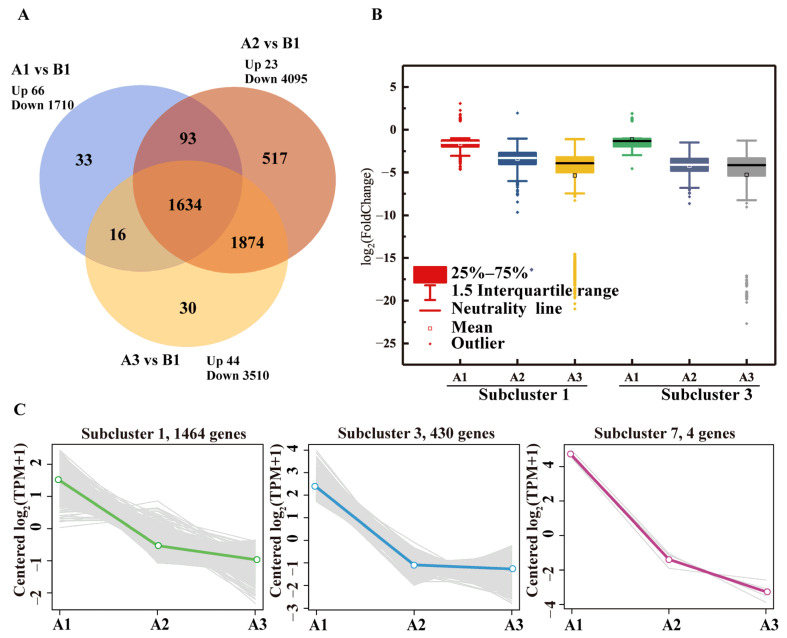
Analysis of differential gene expression in R-001 in response to BPA. (**A**) Venn diagram of gene expression patterns among samples treated with BPA. (**B**) Expression levels of DEGs in subclusters 1 and 3 across the different treatment groups. (**C**) Trends in gene expression patterns of the genes in the downregulated gene clusters.

Under BPA stress, the expression levels of most genes in the R-001 transcriptome were downregulated; only a few genes were upregulated. The number of downregulated genes in each of the three BPA treatment groups was 1710 (A1), 4095 (A2), and 3510 (A3), respectively (Figure 3A). There were significant differences in gene expression profiles among the samples (Figure S1). Groups A2 and A3 exhibited fairly similar gene expression patterns, with 1874 significantly DEGs in common. Although there were fewer significantly decreased DEGs in the A3 group, which had a higher BPA concentration, 376 genes in group A3 were nonetheless downregulated relative to group A2 (Figure 3A and Figure S2). The results showed that the toxicity of BPA severely inhibited gene expression in R-001. This effect was correlated with BPA concentration, and higher concentrations of BPA had a stronger inhibitory effect on gene expression levels in strain R-001.

Under BPA stress, the significantly DEGs in strain R-001 were divided into eight subclusters. A total of 1898 genes were steadily downregulated as BPA concentration increased; these genes were mainly concentrated in subclusters 1, 3, and 7 (Figure 3C). The expression levels of the genes in subcluster 1 decreased an average of 0.324-, 0.094- and 0.024-fold in A1, A2, and A3 treatment groups (Figure 3B). The expression levels of the genes in subcluster 3 decreased an average of 0.461-, 0.054-, and 0.026-fold in A1, A2, and A3 treatment groups (Figure 3B). KEGG annotations indicated that the downregulated genes in these two subclusters were associated with glycolysis/gluconeogenesis, glyoxylate and dicarboxylate metabolism, the pentose phosphate pathway, lipopolysaccharide biosynthesis, and the alanine, aspartate and glutamate metabolism (Figure S3).

#### 3.3.1. Glycolysis/Gluconeogenesis Pathway

**Figure 4:**
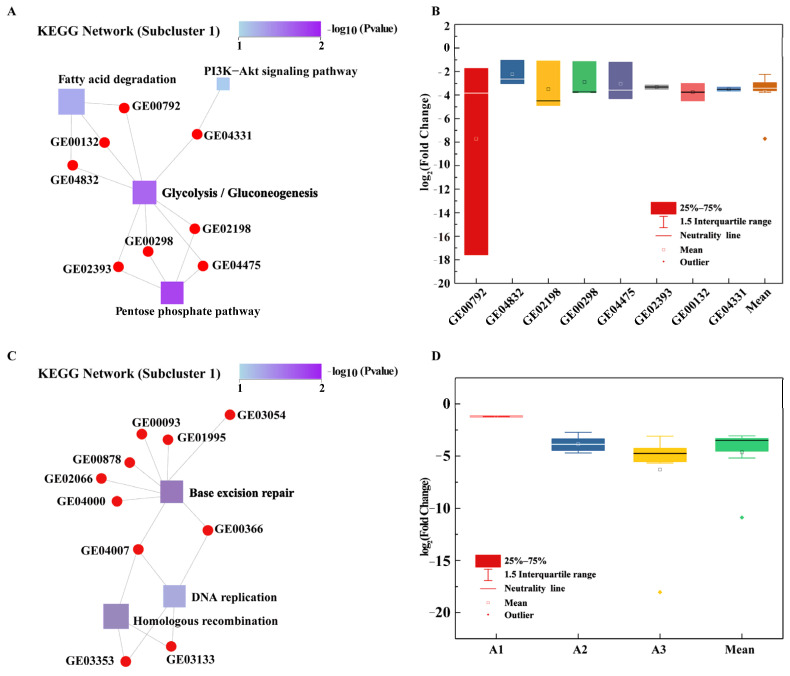
Gene functions and expression levels in strain R-001 exposed to different concentrations of BPA. (**A**) The significantly enriched function-gene interaction network for the glycolytic/gluconeogenic pathway. Square nodes represent functional information, circular nodes represent genes, and lines represent the associations between genes and functions. The color of the square node corresponds to *p* value, with increasing color intensity reflecting an increasing degree of enrichment. The size of the square node corresponds to the number of associated interactions: larger squares interact with more DEGs and thus likely have a greater influence on biological phenomena. (**B**) The expression levels of genes associated with glycolysis/gluconeogenesis. (**C**) The significantly enriched function-gene interaction network for base mismatch repair. (**D**) The expression levels of genes related to the glyoxylate and dicarboxylate metabolism pathway in different BPA treatment groups.

Glycolysis/gluconeogenesis is at the center of the function-gene interaction network. A total of eight genes were associated with this pathway, and these genes were downregulated an average of 0.075-fold (Figure 4A,B). *pck* (gene ID: GE04331, log_2_^FC^ from −3.663 to −3.331) encodes phosphoenolpyruvate carboxykinase, a key enzyme that initiates gluconeogenesis in most bacteria, converting oxaloacetate to phosphoenolpyruvate and CO_2_ [34]. Phosphoenolpyruvate carboxykinase also has a complementary function in some bacteria, catalyzing reverse reactions that are essential for bacterial growth and survival [35]. Studies have shown that the knockdown of *pck* results in growth inhibition in fatty acid media, the accumulation of methylcitrate cycle (MCC) intermediates, and the weakening of tricarboxylic acid (TCA) cycle activity, resulting in strain dormancy [36]. *pgi* (GE02393, log_2_^FC^ from −3.502 to −3.140) encodes a glucose-6-phosphate isomerase that is involved in the upstream glycolysis/gluconeogenesis pathways as well as the conversion of β-D-fructose 6-phosphate to α-D-glucose 6-phosphate, an important precursor of the pentose phosphate pathway [37]. In *Xanthomonas*, mutations in *pgi* lead to a complete blockade of gluconeogenesis, meaning that these bacteria are unable to use pyruvate or intermediates of the TCA cycle for growth [38]. In addition, the loss of *pgi* blocks gluconeogenesis and some hexoses (e.g., sucrose, fructose, and mannose) from entering the pentose phosphate pathway (PPP) or the Entner-Doudoroff pathway. Transgenic strains lacking *pgi* exhibit a 72% reduction in extracellular polymer (EPS) production compared with wildtype strains [39], and decreases in EPS strongly decrease bacterial resistance to toxic substances. *pgm* (GE00298, log_2_^FC^ from −3.771 to −1.151) encodes phosphoglucomutase, which can interconvert D-gucose 1-phosphate and α-D-glucose 6-phosphate. PGM is a key enzyme in the glycolysis pathway and EPS production [40]. Studies have shown that the activity of α-phosphoglucomutase is related to EPS biosynthesis and EPS production, and the inactivation of *pgm* can reduce capsule production in *Streptococcus pneumoniae* [40,41]. *fbp* (GE02198, log_2_^FC^ from −4.499 to −1.105) encodes fructose-1,6-bisphosphatase, a key enzyme in gluconeogenesis that can convert β-D-fructose 1,6-diphosphate to β-D-fructose 6-phosphate [42]. *fbp* null strains are difficult to grow on gluconeogenic carbon sources [42]. *fba* (GE04475, log_2_^FC^ from −4.299 to −1.215) encodes fructose-bisphosphate aldolase, which is also central to glycolysis/gluconeogenesis and which catalyzes the cleavage of fructose 1,6-diphosphate to glyceraldehyde 3-phosphate and dihydroxyacetone phosphate [43]. These results showed that many genes involved in glycolysis/gluconeogenesis were downregulated in strain R-001 in response to BPA stress. This may result in the inability of intermediates to enter the PPP and TCA cycles, producing insufficient energy for R-001 growth and weakening TCA cycle activity. This process may eventually lead to reductions in thallus activity and damage. In addition, the downregulation of *pgi* and *pgm* may lead to the reduction of EPS secretion in strain R-001, further reducing the ability of the strain to resist environmental toxins.

#### 3.3.2. Glyoxylate and Dicarboxylate Metabolism

Glyoxylate and dicarboxylate metabolic pathways were significantly enriched in the KEGG annotations of subcluster 3 (Figure 4B). The glyoxylate and dicarboxylate metabolism is the primary mechanism of material metabolism and energy supply in bacterial cells [44]. Genes in this pathway tended to be downregulated, with an average log_2_^FC^ value of −3.776. The average log_2_^FC^ values for genes in this subcluster at different concentrations of BPA were −1.201 for group A1, −3.848 for group A2, and −6.28 for group A3 (Figure 4D, Table S7). Studies have shown that biochar induces the upregulation of genes in the glyoxylate and dicarboxylate metabolism, the citric acid cycle, and other metabolic pathways and that this upregulation is conducive to the bacterial biodegradation of 2,2’,4,4’-tetrabrominated diphenyl ether [44]. Glyoxylate and dicarboxylate metabolic pathways may also play a role in the degradation and detoxification of HCBD by *Rhodopseudomonas palustris* YSC3 [45].

In general, various concentrations of BPA have toxic effects on R-001, primarily due to the BPA-driven downregulation of genes in the glycolysis/gluconeogenesis, glyoxylate and dicarboxylate, and pentose phosphate metabolism pathways. This downregulation reduces the biological flux of intermediates, strain energy levels, and the production of EPS, leading to decreases in strain activity and toxicity resistance.

### 3.4. Resistance of R-001 to BPA Toxicity

#### 3.4.1. Downregulation of BER-Related Genes to Increase Strain Mutation

**Figure 5:**
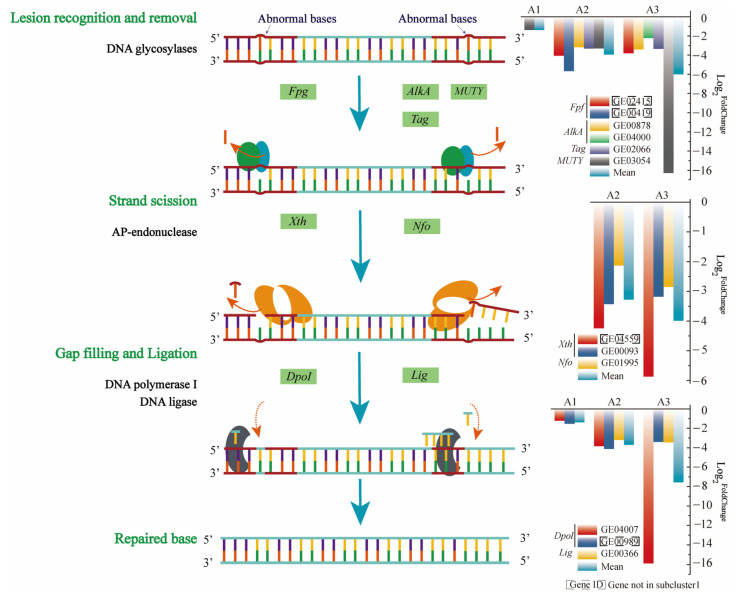
DNA excision repair mechanisms and gene expression levels in R-001 under BPA stress. A dashed border around a gene symbol indicates that this gene did not belong to subcluster 1 but was involved in the DNA excision repair process.

A study has shown that the downregulation of proteins involved in DNA mismatch repair can completely inhibit the mismatch repair mechanism of *Escherichia coli*, leading to an increase in the frequency of strain mutations, which may lead to improvements in the resistance of this bacterium to the toxic damage caused by BPA [46]. Therefore, the expression levels of genes participating in DNA damage repair were analyzed in strain R-001. In the BPA-treated group, base excision repair was significantly enriched in subcluster 1 (Figure S3A). This function was located at the central node of the gene-function interaction network and was associated with a total of eight genes (Figure 4C). Base excision repair (BER) is the primary mechanism used to remove abnormal bases, such as oxidized bases and alkylated bases, and BER usually repairs bases with minimal damage [47]. BER can be divided into four steps: recognize, remove, resynthesize, and religate (Figure 5).

First, DNA glycosidases encoded by *alkA* (GE00878), *tag* (GE02066), *mutY* (GE03054), and *fpg* (GE02415) recognize the damaged base, excise the glycosidic bond and generate the abasic (AP) site [48]. Second, the phosphodiester bond is hydrolyzed at the 5’-terminus to dealkalize deoxyribose using exonuclease III, encoded by *Xth* (GE04559), or endonuclease IV, encoded by *Nfo* (GE01995) [49]. This dealkalization produces 3’-OH and 5’-deoxyribo-phosphate terminus (5′drp), creating a gap in the DNA [47]. Finally, DNA polymerase I, encoded by *DopI* (GE04007, GE00989), and DNA ligase, encoded by *Lig* (GE00366), fill the gap to complete the repair process [47] (Figure 5). Consistent with previous findings, genes involved in base repair were significantly downregulated under BPA stress. The mean log_2_^FC^ values of the genes encoding DNA glycosidase were −1.37 (A1), −3.928 (A2), and −5.982 (A3) at the three BPA concentrations, respectively. The genes encoding exonuclease and endonuclease were not significantly differentially expressed at 5 mg/L BPA, but were downregulated at BPA concentrations of 15 mg/L and 40 mg/L, with mean log_2_^FC^ values of −3.27 and −3.977, respectively. The average decrease in the expression levels (log_2_^FC^) of the genes encoding DNA synthase at the three concentrations were −1.38 (A1), −3.975 (A2), and −9.635 (A3), respectively. At 15 and 40 mg/L BPA, the average decrease in the expression levels (log_2_^FC^) of the genes encoding DNA lintase were −3.2 and −3.44, respectively (Figure 5).

Base excision repair plays an important role in the maintenance of bacterial DNA integrity, and the deletion of BER-related genes increases bacterial mutation rates [47]. For example, BER loss leads to increased spontaneous mutagenesis in *Bacillus subtilis* [50]. Therefore, the downregulation of BER-related genes in strain R-001 in response to BPA stress increases the mutation rate of this strain, thereby improving resistance to the toxic damage caused by BPA; toxicity resistance was positively correlated with BPA concentration.

#### 3.4.2. Upregulation of Metabolic Genes to Maintain Energy Supply

**Figure 6:**
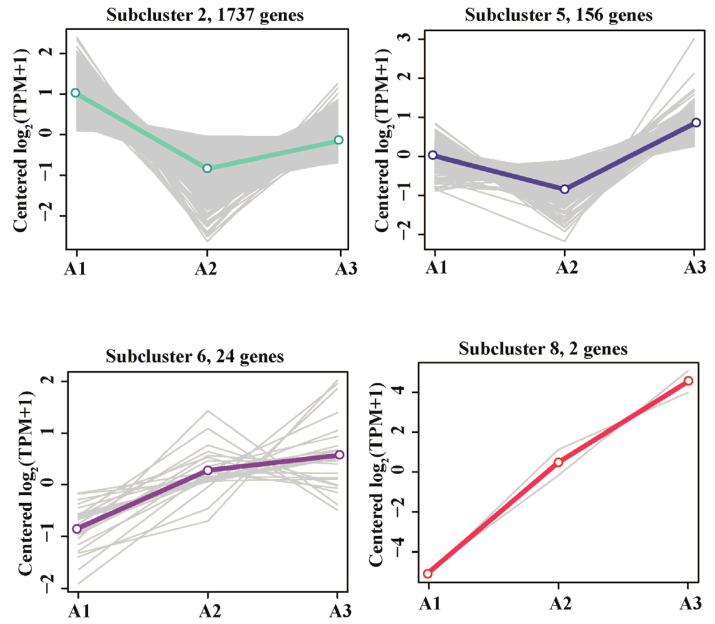
Trends in the expression patterns of upregulated DEG clusters at different concentrations of BPA.

Across the other gene subclusters, the expression patterns of 1919 genes exhibited a “V” shape or tended to increase continuously (Figure 6). Gene functional annotations were very similar in the A1 and A3 treatment groups, with both groups enriched in the following pathways: fatty acid metabolism; fatty acid degradation, pyruvate metabolism; valine, leucine, and isoleucine degradation; propanoate metabolism; and carbon metabolism (Figure S4A,C). The upregulation of genes in these metabolic pathways may provide additional energy to compensate for the loss of energy caused by the toxic effects of BPA. First, *fadA,* encoding acetyl-CoA acyltransferase, was the most significantly upregulated gene in the fatty acid degradation pathway (log_2_^FC^ = 6.6). Studies have shown that *fadA* is one of the key genes in the metabolism of 2,4,6-trichlorophenol (2,4,6-TCP) by activated sludge; *fadA* converts intermediates into acyl-CoA or succinyl-CoA, and the final metabolites enter the TCA cycle to provide energy for strain growth [51]. Second, the TCA-cycle genes *gltA*, *icd*, *sucB*, *sdhA*, *fumC,* and *moo* were upregulated when the concentration of BPA was 40 mg/L, and this upregulation led to an increased energy supply, improving cellular activity levels [52]. In addition, when BPA concentration increased from 15 mg/L to 40 mg/L, several genes in the oxidative phosphorylation pathway were significantly upregulated (e.g., *sdhA* and *ppk1*; Table S8), possibly increasing ATP synthesis. This increasing trend in gene expression level was consistent with the increase in the ATP content of the bacterium (Figure 2D). Genes encoding acetate kinase, methylmalonyl-CoA mutase, and pyruvate carboxylase, all of which play important roles in energy production in bacteria [53,54,55], were also upregulated (Table S8).

These results showed that the inhibitory effects of BPA on the metabolic genes were limited. Strain R-001 compensated for the BPA-driven loss of energy from glycolysis/gluconeogenesis and the glyoxylate and dicarboxylate metabolic pathway by upregulating genes in other energy-related metabolic pathways, generating additional intermediates and energy to maintain metabolic activity.

#### 3.4.3. Upregulation of Transport-System Genes to Maintain Cell Homeostasis

ABC transporters were annotated in the A1 and A2 treatment groups (Figure S4A,B), but the annotated genes belonged to different transport systems. In the A1 group, the upregulated genes (*opuBB* and *opuBC*) belonged to the osmoprotectant uptake (Opu) system, which maintains cellular physiological function through the uptake of a variety of compatible solutes (e.g., choline and glycine betaine aldehyde) [56,57]. *opuBC* (GE02040) encodes a substrate-binding protein in the osmoprotection transport system. This extracellular solute receptor is immobilized on the outer surface of the cytoplasmic membrane via the lipid modification of the N-terminal Cys residue. *opuBB* (GE02043) encodes the permease protein in the osmoprotective transport system. The significant upregulation of two important genes in the Opu system (Table S9) suggests that strain R-001 may regulate osmolality to maintain cell stability in response to BPA toxicity. Similarly, P450-carrying *Escherichia coli* may also respond to BPA toxicity by modulating osmolality [46]. In the A2 group, the upregulated genes encoded ABC transporters that are responsible for the transport of iron, which is an essential nutrient for bacterial biological metabolism. *fhuD* (GE00670) encodes a substance-binding protein in the iron-complex transport system, which transports iron pigments to permeases. The iron complex transport system permease encoded by *fhuB* (GE00669) interacts with FhuD to transport siderophores into the cytoplasm, a process that is mediated by ATP hydrolysis in FhuC [58]. Our results thus suggested that strain R-001 may adopt a variety of transport mechanisms to mitigate the toxic effects of BPA, including regulating osmotic pressure or iron complex transport to maintain cellular activity.

### 3.5. Genes and Pathways Associated with BPA Degradation in Strain R-001

During the degradation of BPA by R-001, there were no significant differences in the expression levels of the BPA-degradation genes in the treatment group. However, the BPA-degradation genes were upregulated from 1.56- to 19.35-fold in the high-BPA group as compared to the low-BPA group. After exposure to various concentrations of BPA, the qPCR analysis showed that the expression levels of the BPA-degradation genes were 1.48- to 2.95-fold greater than the expression level of the reference gene (*recA*; Table 1). In particular, cytochrome P450 plays a critical role in the degradation of BPA [59,60,61], and the addition of cytochrome P450 inhibitors will inhibit the degradation of BPA in different degrees [62]. qPCR analysis showed that the gene GE00504, which encodes P450 monooxygenase, was upregulated an average of 1.67-fold after induction. The amino acid sequence of GE00504 had 29.26% and 32.05% identity with the amino acid sequences of cytochrome P450 monooxygenase from *Sphingomonas* sp. AO1 (BAG15884.1) and *Bacillus* sp. GZB (OMQ03826.1), respectively. In addition, studies have shown that multicopperoxidase (laccase) can also complete the biotransformation of a variety of phenolic substances [63]. The gene GE00283, encoding multicopperoxidase, was upregulated an average of 2.36-fold after induction, and the amino acid sequence of GE00283 had 29.38% identified with laccase from *Bacillus* sp. GZB (APF29085.1). Recombinant laccase completely degrades BPA and has certain detoxification effects [64]. The upregulation of the GE00283 gene at a BPA concentration of 40 mg/L may also contribute to the BPA detoxification abilities of R-001. In addition, we also detected the upregulated expression of *hqdD* (GE03927, Tables S3–S5), which can encode Maleylacetate reductase. Kolvenbach et al. have confirmed that Maleylacetate reductase can transform maleylacetate into 3-oxoadipate, both of which can be obtained by further degradation of hydroquinone [65,66]. Although these intermediates were not found in our LC-MS results, it can still be predicted that *hqdD* can participate in the subsequent biodegradation of BPA.

**Figure 7:**
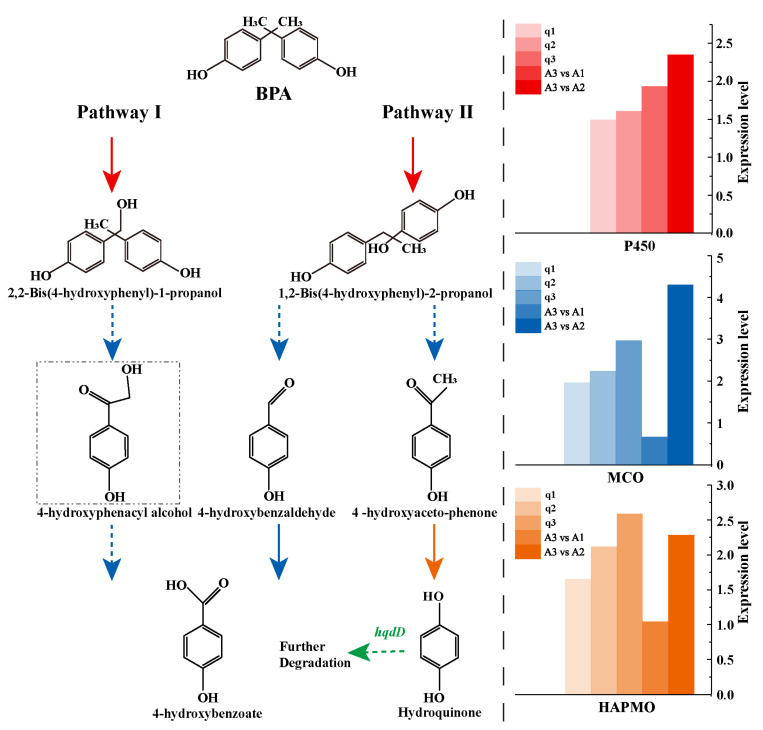
The putative BPA degradation pathway of strain R-001. 4-hydroxyphenacyl alcohol was not detected in LC-MS.

Based on the above analysis and LC-MS results (Figure S5), we found that R-001 degraded BPA via two pathways (Figure 7). In pathway I, P450 catalyzes the hydroxylation of BPA methyl to produce 2,2-bis(4-hydroxyphenyl)-1-propanol (2,2-BIS). The product is finally cleaved to 4-hydroxybenzoate by multicopperoxidase [27]. In pathway II, cytochrome P450 acts on the quaternary carbon atom of BPA and undergoes hydroxylation to form 1,2-bis(4-hydroxyphenyl)-2-propanol, which is followed by oxidation and cleavage in the presence of MCO to form 4-hydroxyacetophenone and 4-hydroxybenzaldehyde. The former product is the main intermediate in the BPA degradation process [27] and has been previously used to maintain the growth and metabolism of BPA-stressed bacterial strains [62]. After induction by 4-hydroxyacetophenone, the gene encoding 4-hydroxyacetophenone monooxygenase (EC:1.14.13.84) was significantly upregulated (Table 1), which promoted further cleavage of the intermediate to form 4’-hydroxyacetophenone.

## 4. Conclusions

BPA pollution poses an increasingly serious problem. Although some BPA-degrading microorganisms have been isolated and identified, the toxic effects of BPA on the degradation abilities and toxicity-resistance mechanisms of these bacteria remain unclear. Our results demonstrated that *Rhodococcus equi* DSSKP-R-001 had a high ability to degrade BPA and tolerated 40 mg/L BPA while maintaining BPA degradation performance. However, due to the toxic effects of BPA, the growth, metabolism, and gene expression of strain R-001 were inhibited. The OD value, total protein content, and ATP content of the strain R-001 exhibited a “V”-type relationship with BPA concentration: these parameters decreased at BPA concentrations of 5–15 mg/L and slowly increased at concentrations of 15–40 mg/L. In addition, 1794 genes were downregulated in strain R-001 in response to BPA stress, and most of these genes were related to energy metabolism. Nonetheless, strain R-001 exhibited some resistance to BPA toxicity. This strain mitigated BPA-associated toxic damage by regulating the expression levels of genes related to base excisional repair, energy metabolism, osmoprotection, and the iron complex transport system. In addition, the genes encoding cytochrome P450 monooxygenase and multicopperoxidase were upregulated during BPA degradation. Moreover, the similarities between the amino acid sequences of these upregulated genes and known proteins suggested that the upregulated genes might play an important role in the initial step of BPA degradation by R-001, converting BPA into small molecule intermediates to complete the detoxification process. This study expanded our knowledge of BPA-degrading microorganisms, clarified the details of the toxicity-resistance and BPA-degradation mechanisms of *Rhodococcus*, and provided a theoretical basis for the application of *Rhodococcus* to BPA bioremediation.

## Figures and Tables

**Table 1:** BPA-degrading genes in strain R-001.

Gene ID	Gene Description	Synonym	qPCR			Log_2_^FC^	
			q1	q2	q3	A3 vs. A1	A3 vs. A2
GE00504	Cytochrome P450 130	P450	1.48	1.60	1.92		2.337
GE00283	Multicopper oxidase	MCO	1.93	2.21	2.95	0.64	4.28
GE03924	4-hydroxyacetophenone monooxygenase	HAPMO	1.64	2.10	2.57	1.027	2.273

## Data Availability

The raw sequence data has been submitted to the NCBI database and accession number is PRJNA842860.

## Supplementary Materials

The following supporting information can be downloaded at: https://www.mdpi.com/article/10.3390/microorganisms11010067/s1, Figure S1: PCoA principal coordinates analysis for treated samples; Figure S2: Heat map (A) and volcano map (B) of distance between samples of different treatment groups; Figure S3: KEGG functional annotation results of genes in subcluster 1 (A) and subcluster 3 (B); Figure S4: KEGG functional annotation of up-regulated differentially expressed genes in strains exposed to different concentrations of BPA; Figure S5: Intermediate products of BPA degradation by R-001: Table S1: 5 mg/L BPA-treated group (A1) significantly differentially expressed genes; Table S2: 15 mg/L BPA-treated group (A2) significantly differentially expressed genes; Table S3: 40 mg/L BPA-treated group (A3) significantly differentially expressed genes; Table S4: A3 vs A2 group significantly differentially expressed genes; Table S5: A3 vs A1 group significantly differentially expressed genes; Table S6: Primer information; Table S7: Expression levels of genes in sbucluster 3 annotated to Glyoxylate and dicarboxylate metabolism pathway; Table S8: Differentially expressed genes related to energy metabolism; Table S9: Differentially expressed genes related to ABC transporters.
