# Supplementary material for: Mechanisms of BPA Degradation and Toxicity Resistance in Rhodococcus equi
Source: Microorganisms. 2022 Dec 26;11(1):67. doi: 10.3390/microorganisms11010067 (PMC9862853; doi:10.3390/microorganisms11010067)
Supplement: Supplementary file 1 [file microorganisms-11-00067-s001.zip › Supplementary materials.pdf]

Mechanism of BPA degradation and toxicity resistance in *Rhodococcus equi*

Kejian Tian[a], Yue Yu [a], Qing Qiu [a], Xuejian Sun [a], Fanxing Meng [b], Yuanping Bi [c], Jinming Gu[a], Yibing Wang[a], Fenglin Zhang[a], Hongliang Huo [a,d,e,*]

a School of Environment, Northeast Normal University, No. 2555 Jingyue Avenue, Changchun City, Jilin Province, China

b Jilin Province Water Resources and Hydropower Consultative Company of PR China, Changchun City, Jilin Province, China

c School of Life Sciences, Northeast Normal University, No. 5268, Renmin Main Street, Changchun City, Jilin Province, China

d Jilin Province Laboratory of Water Pollution treatment and Resource Engineering, Changchun, 130117, China

e Northeast China Low Carbon Water Pollution treatment and Green Development Engineering Research Center

Content:

Figures : 5

Figure S1. PCoA principal coordinates analysis for treated samples.
Figure S2. Heat map (A) and volcano map (B) of distance between samples of different treatment groups.
Figure S3. KEGG functional annotation results of genes in subcluster 1 (A) and subcluster 3 (B).
Figure S4. KEGG functional annotation of up-regulated differentially expressed genes in strains exposed to 5 mg/L (A), 15 mg/L (B) and 40 mg/L (C) of BPA.
Figure S5. Intermediate products of BPA degradation by R-001.

Tables : 9 (in Excel)

Table S1. 5 mg/L BPA-treated group (A1) significantly differentially expressed genes
Table S2. 15 mg/L BPA-treated group (A2) significantly differentially expressed genes
Table S3. 40 mg/L BPA-treated group (A3) significantly differentially expressed genes
Table S4. A3 vs A2 group significantly differentially expressed genes
Table S5. A3 vs A1 group significantly differentially expressed genes
Table S6. Primer information
Table S7. Expression levels of genes in sbucluster 3 annotated to Glyoxylate and dicarboxylate metabolism pathway
Table S8. Differentially expressed genes related to energy metabolism
Table S9. Differentially expressed genes related to ABC transporters

1. Supplementary Figures

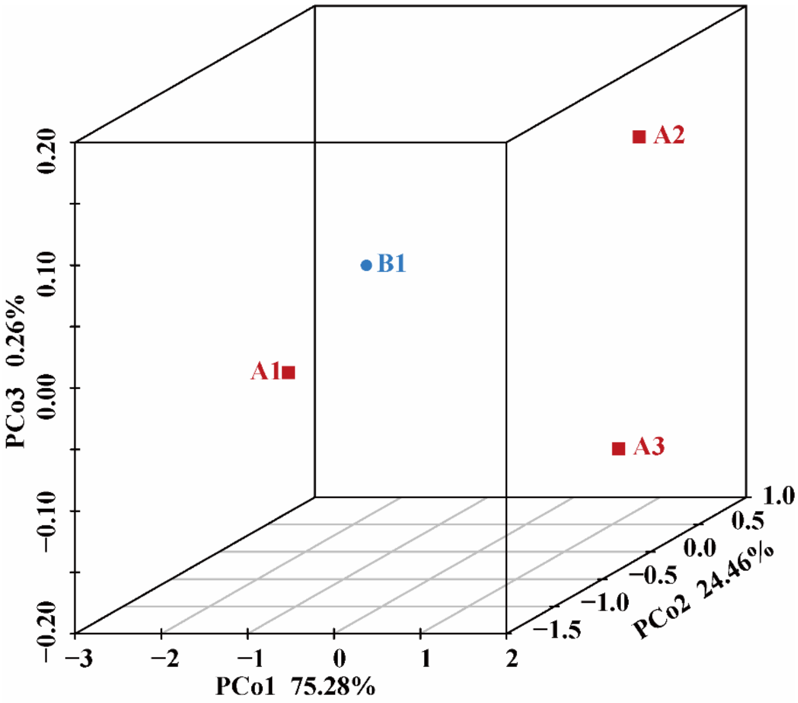

Figure S1. PCoA principal coordinates analysis for BPA treated samples.

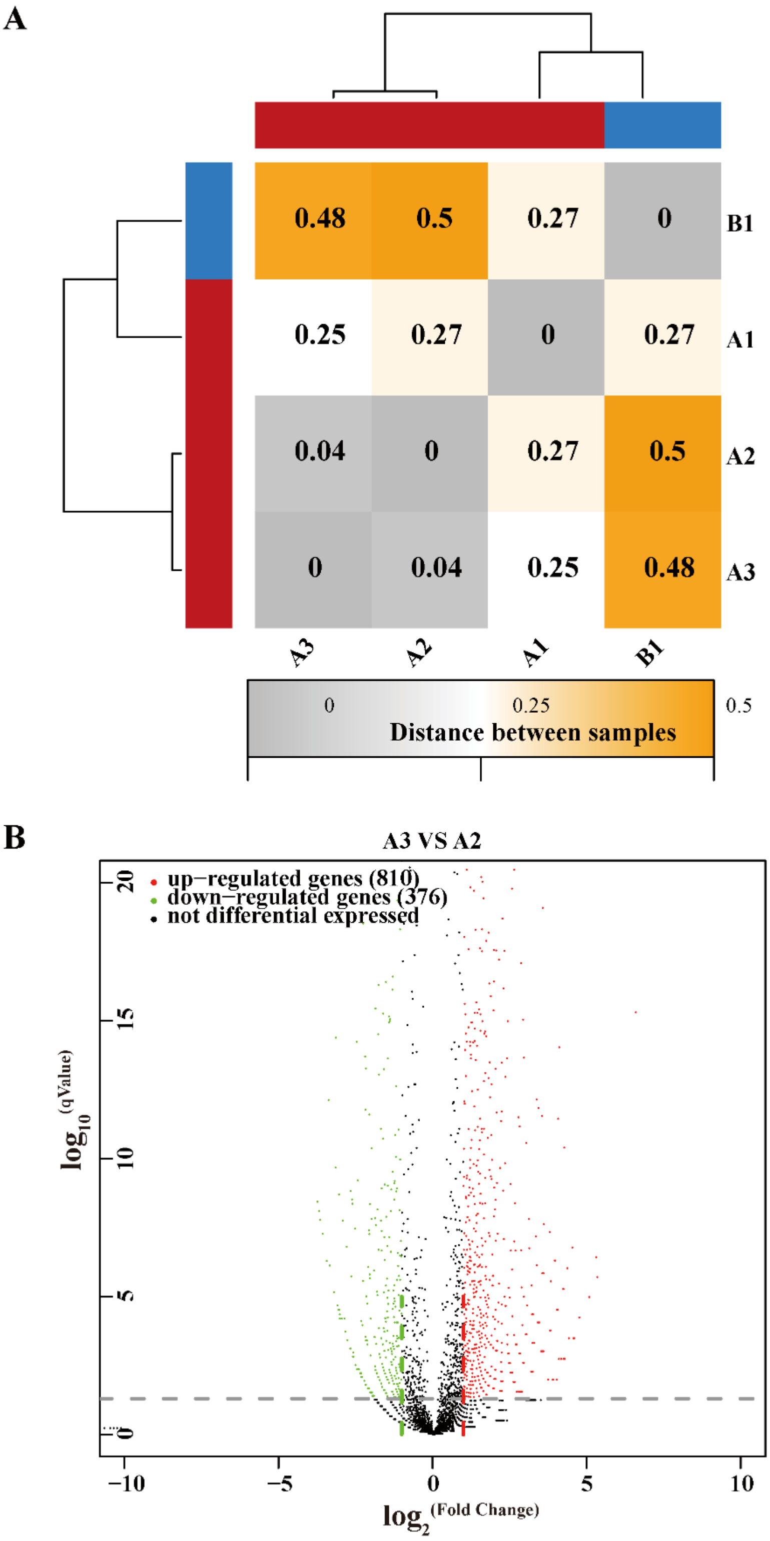

Figure S2. Heat map (A) and volcano map (B) of distance between samples of different treatment groups. Color blocks represent distance values, gray indicates high similarity between samples, and yellow indicates low similarity between samples.

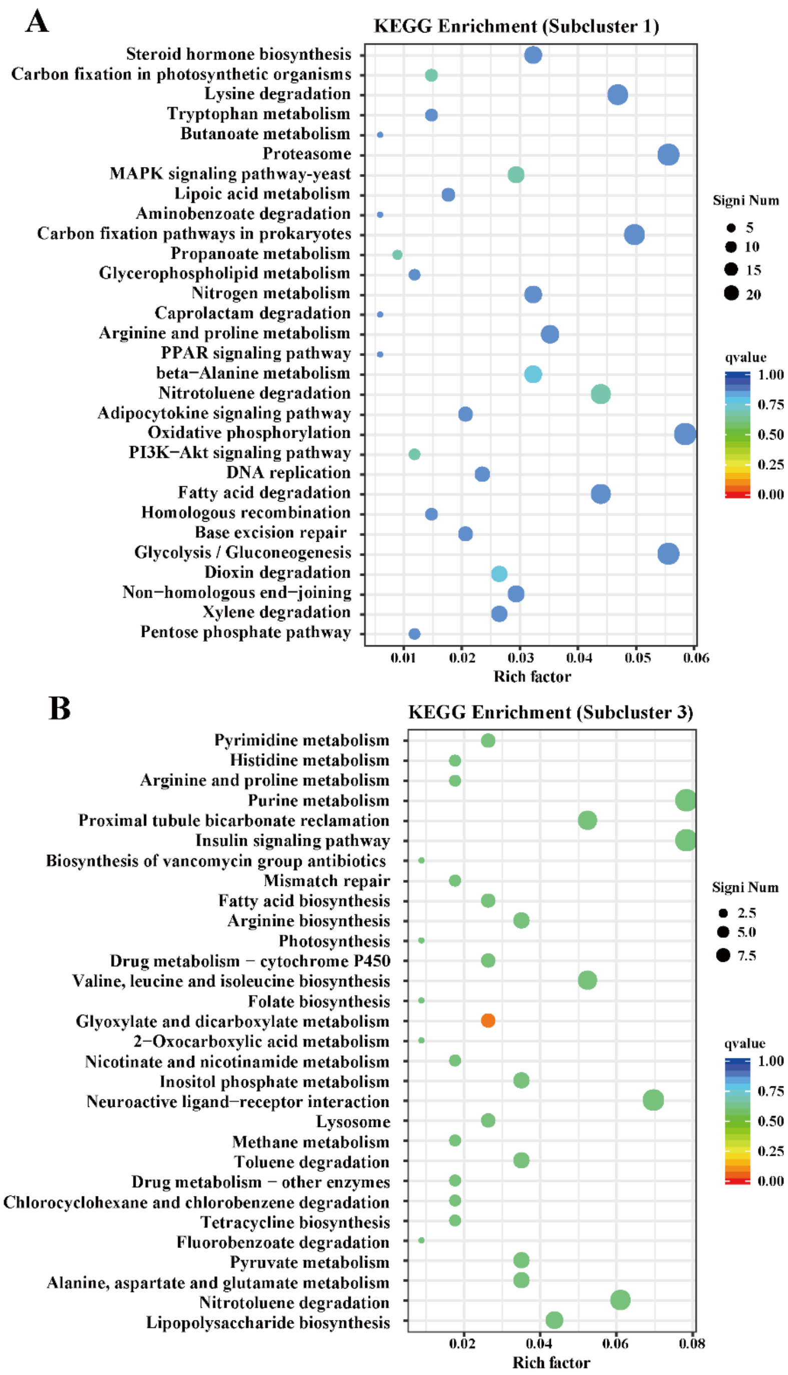

Figure S3. KEGG functional annotation results of genes in subcluster 1 (A) and subcluster 3 (B).

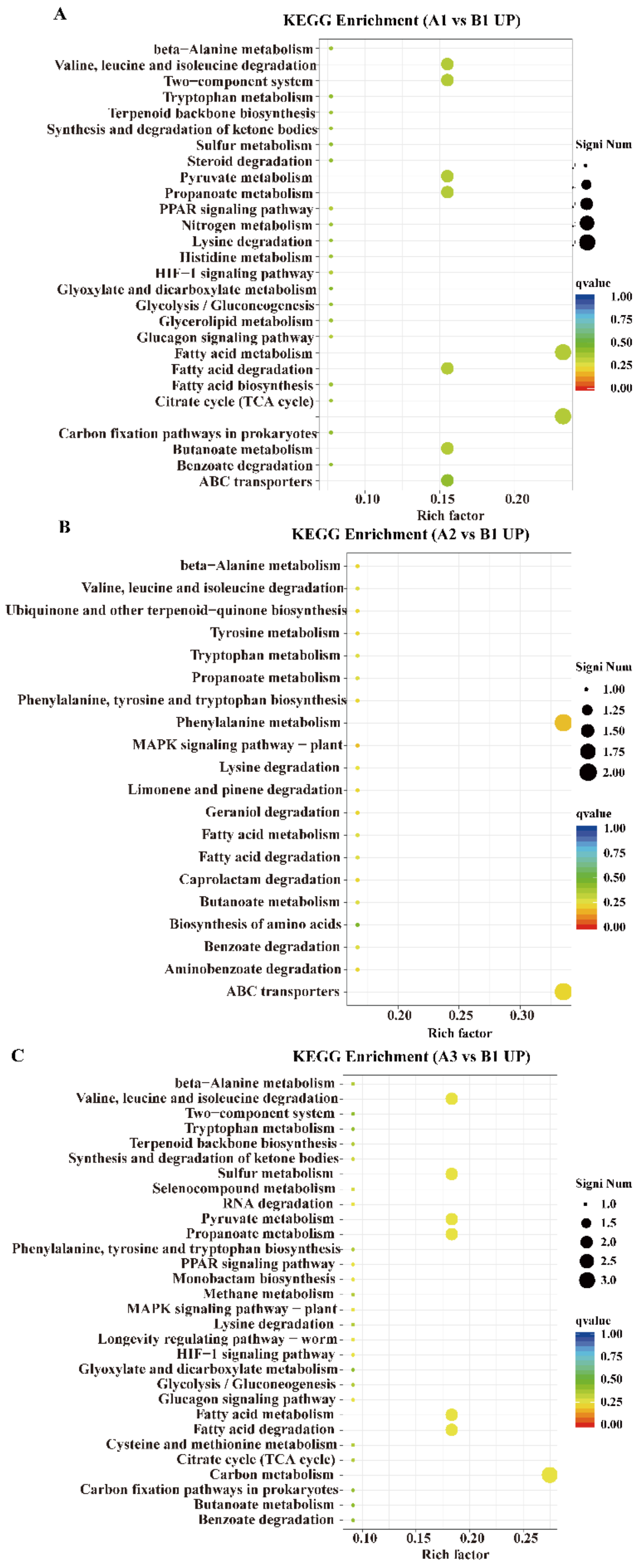

Figure S4. KEGG functional annotation of up-regulated differentially expressed genes in strains exposed to 5 mg/L (A), 15 mg/L (B) and 40 mg/L (C) of BPA.

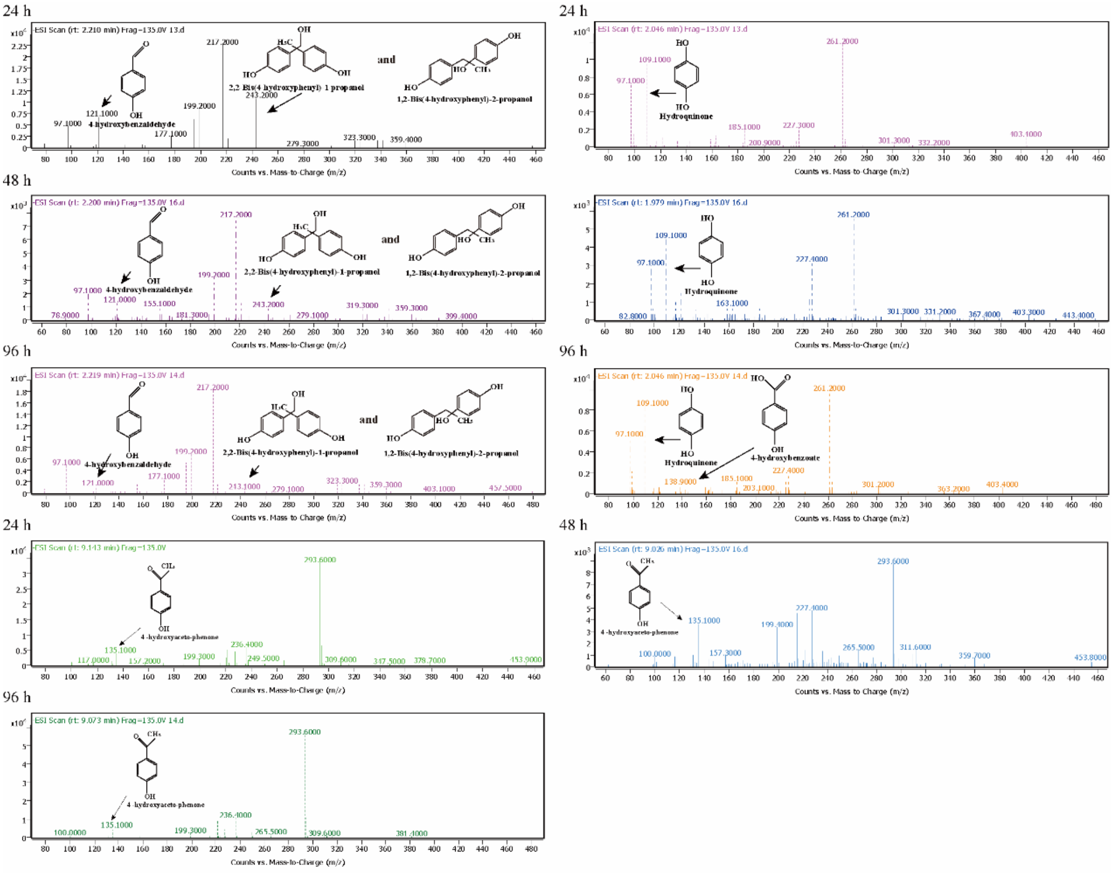

Figure S5. Intermediate products of BPA degradation by R-001.
